# Blockchain in Health Care: Hope or Hype?

**DOI:** 10.2196/17199

**Published:** 2020-07-10

**Authors:** Rania El-Gazzar, Karen Stendal

**Affiliations:** 1 University of South-Eastern Norway Hønefoss Norway

**Keywords:** blockchain, health care, innovation, security, implications

## Abstract

There has been an increasing interest in blockchain technology from the health care sector in the last couple of years. The value proposition for using blockchain technology in the health care sector is to share sensitive patient data among health care entities securely and to empower patients. Blockchain technology allows patients to have an active role in developing and updating their own patient data. However, is blockchain technology really the silver bullet it seems to be? With this paper, we aim to understand the benefits and challenges of blockchain technology in the health care sector. We discuss innovation and security implications concerning blockchain technology in health care. Furthermore, we show that there is a need for more use cases to ensure the secure sharing of data within the health care sector. In our opinion, blockchain technology will not solve the issues encountered by the health care sector; in fact, it may raise more issues than it will solve.

## Introduction

Beyond its first application as the peer-to-peer payment system Bitcoin, blockchain technology is anticipated to revolutionize industries and sectors [1,2]. The implementation of blockchain technology has been clearly dominant in the financial industry [3], the supply chain industry [4], the payments industry [5,6], and e-commerce [7]. The health care sector can potentially benefit from blockchain technology by making health care information systems patient-centric and facilitating health data–sharing securely and efficiently [8]. A plethora of studies have proposed various potential use cases for using blockchain in health care [8-10]; however, the vast majority of those proposed use cases were not implemented [10]. In their recent literature review, Hasselgren et al [8] analyzed 39 studies on blockchain in health care that proposed solutions that were implemented as proof of concept.

The common use cases that benefit from blockchain-based solutions for a patient-centric health care information system include patient-managed health records, enhanced insurance claim processes, enhanced health care research, and advanced medical records shared among patients and health care providers [11]. Despite the suitability of blockchain solutions for problems and innovation needs in health care information systems, the feasibility of fully implementing those solutions is scarce to moderate [8]. Even if some solutions have proven feasible to implement into practice, they require reductions in data size and operating costs, as well as better protection of personal information to maintain privacy and security [12].

Further, barriers to the feasibility of fully implementing blockchain-enabled patient-centric electronic medical records include interoperability and scalability issues [9]. Interoperability issues manifest in the lack of standards among various blockchain-based solutions [9]. However, proposals have been introduced to address those issues [8]. Due to the high volume of clinical data, scalability issues arise, as blockchain-based solutions have data size limitations [9,13]. Patient engagement seems to be a benefit of blockchain-based solutions in health care; however, it is likely not the case for all types of patients, as not all patients are enthusiastic about managing their own data [9].

Blockchain-based solutions have not been adequately assessed for their compliance to individuals’ rights protected by the General Data Protection Regulation (GDPR) [8,14]. The abovementioned opportunities, barriers, and concerns have implications for patients, health care providers, and researchers. Furthermore, the legal, security, and privacy implications deserve further exploration. This may lead to some workarounds to make the blockchain architecture GDPR compliant, which have not yet resulted in something concrete [14-16].

Through this viewpoint article, we aim to discuss innovation and security implications that blockchain brings to the health care sector. We did not attempt to conduct a thorough literature review on the topic, as there are already comprehensive literature reviews on blockchain in health care [8-10,14]. Rather, we discuss our viewpoint on the implications of blockchain for the health care sector, with support from the current body of literature.

Thus, we aim to answer our research question: What implications may blockchain technology bring to the health care sector?

First, we present a background on blockchain technology. Second, we discuss implications of innovating with blockchain in health care. Third, we discuss the security implications of blockchain in the health care sector. Fourth, we summarize our discussion, and finally, conclude this paper.

## Background of Blockchain Technology

Blockchain technology is described as a disruptive innovation that brings opportunities and challenges to various industries and sectors, and it deserves further exploration [17-19]. There is an ongoing debate as to which came first, blockchain or Bitcoin [1,2,20]. Blockchain is the underlying technology with broader capabilities and characteristics. Bitcoin is just an application area for trading that inherits blockchain characteristics [21]. Stuart Haber and W Scott Stornetta invented the notion behind blockchain first, when they proposed a framework for a “timestamping digital document” to calculate hash values that uniquely identify documents and save them in certificates with a timestamp [22]. These documents are linked by a data structure with the hashes of previous records. Nakamoto [2] adopted the framework proposed by Haber and Stornetta, creating the first Bitcoin peer-to-peer payment system based on timestamped blocks of transactions, which are chained using the hash values of previous blocks. Bitcoin then became commonly known as a means for trading with cryptocurrency [23].

Swan [13] defined blockchain as a decentralized transparent ledger with transaction records. Blockchain contains a set of data blocks, each of which contains data on multiple transactions (ie, transactions list, timestamp, nonce, hashes of the transactions and their root hash or block hash, and the hash of the previous block). As more blocks are added to the chain, the distributed ledger becomes a complete transaction history book [24]. Before adding the new transactions to the ledger, the consensus mechanism is applied by multiple participants to validate the transaction and the block. Transactions reside in the block for a specified time until the consensus process is done. Then, the block of transactions is stored in the ledger, where the information cannot be changed [24]. If the hash of a block is modified, the block is no longer valid [25], which makes subsequent blocks invalid as well, and this will require verifying the block after recalculating its hash and the hashes of subsequent blocks [26].

There are two basic deployment forms of blockchain; these are public permissionless and private permissioned blockchains [1,27]. Public permissionless blockchains are open and decentralized, where anyone can join and leave the network as reader and writer at any time (eg, Bitcoin). The network has no central authority to monitor it and no one owns and controls the network. Private permissioned blockchains only authorize a limited set of readers and writers (eg, Hyperledger). The network has a central authority that assigns the right to individuals to read and write operations.

Several definitions of blockchain mainly refer to the characteristics of public permissionless blockchains, such as absolute immutability, anonymity, decentralization in running the consensus mechanism, and openness [1,21,26]. The definitions do not provide a description of private permissioned blockchains, which are managed by a central trusted authority that controls the consensus process, in which the identities of participants are predefined and access permissions are restricted [28].

Blockchain technology is claimed to be an “accelerating force of innovation” that promises a wide range of benefits [17]. However, the claims about blockchains being tamperproof and offering strong security are challenged by a long list of security threats [29]. Blockchains are claimed to be immutable and unable to be hacked, but this has been proven invalid [30,31]. Furthermore, blockchains are energy-consuming, which entails considerable costs (eg, network performance problems) [25,32]. This poses a concern about whether the benefits and promises brought by blockchain can be taken for granted or whether they will become a threat to the ambitions for innovation and better security. Consequently, practice and academia still have questions to address regarding benefits and risks that arise from blockchains, including whether blockchain is a radical or incremental innovation in nature [17].

## Innovation and Security Implications of Blockchain in Health Care

### Innovation and Security Needs

Whether blockchain is a blessing or a curse, in terms of innovation and security, it is a matter of what it adds to a no-blockchain situation. It can add information technology (IT) or business costs or complexity [33]. An ever-present challenge for any industry or sector is the balance between implementing modern IT solutions and keeping information assets safe from security threats. Blockchain technology emerged with the promise to address this challenge; it enables innovation by implementing a modern decentralized information infrastructure [17,19,34].

The health care sector has a long history of heavy regulation and bureaucratic inefficiency that has decelerated its innovation [35-37], and an increasing number of data breaches have been reported in recent years [38]. It is claimed that innovating with blockchain ensures the privacy and security of highly vulnerable and sensitive patient data in the cyber world [35]. However, there are more experiments of proposed blockchain solutions than full implementations in the health care sector [36,39,40].

### Implications of Innovating With Blockchain in Health Care

Blockchain is a disruptive innovation that can leverage health care information systems’ abilities to improve patient care; however, this has considerable regulatory, financial, and operational implications [41,42]. Private permissioned blockchains are a proper option for the health care sector to deal with sensitive patient data [8,27]. This type of blockchain deployment has beneficial implications for the use of blockchain in health care. The use cases suggested by researchers for blockchain in health care include patient-managed medical records, improved insurance claim processes, accelerated medical research with the use of shared anonymous patient data, and an advanced health data ledger maintaining clinical transaction logs, pharmaceutical supply chains, and consent recording [8,11,43,44].

The exploration of blockchain’s compliance with GDPR is scarce despite its importance [8]. Private permissioned blockchains have implications for GDPR due to the central authority controlling the network and access to personal data [45]. In the case of using patient data to support health care researchers [11], a pseudonymization technique is required to protect patients’ sensitive data [16], which may pose a risk of reidentification (ie, linking the pseudonym code or metadata to the patient’s health data), raising a conflict with GDPR [45]. This requires a careful consideration of the use case and the design of the blockchain-based health information system [46].

Blockchains are immutable; therefore, it is not possible to delete a block. Thus, blockchain does not comply with the requirement under GDPR that stipulates that data subjects have the right to request for their data to be erased, including health sensitive data [9,47]. A proposed workaround is to store the patient data off-chain and have the pseudonym codes stored on-chain [16,47]. However, this implies that the pseudonym code and any transaction records on the patient data that are stored on-chain would still be existent even after deleting the patient data that were stored off-chain [47]. To reverse the immutability of blockchain, a proof-of-concept prototype for a “forgetting blockchain” was proposed to delete old data from private permissioned blockchains; however, the prototype still has limitations to address [15].

Beck and Müller-Bloch [19] argued that blockchain is a radical innovation that outdates the conventional distributed systems approach with a different architecture and characteristics [19,48]. Thus, radical innovations are difficult to implement and they bring more complex challenges, which require organizational readiness and the updating of old organizational knowledge and IT infrastructure [49]. This has financial implications for using blockchain technology in the health care sector; despite the fact that it has the potential to improve the quality of medical services, it may create financial uncertainties [41]. The top challenges facing the adoption of blockchain in health care include computational overhead, lack of interoperability and standardization, privacy concerns, and the uncertainty about who is responsible for the cost of technology implementation and who profits from it [14,50]. Barriers to adopting blockchain in the health care sector include immaturity of the technology itself, insufficient skills to understand and implement it, lack of buy-in, and lack of clear return on investment [51]. The lack of buy-in goes back to the unfamiliarity of blockchain, the negative attitudes of medical doctors toward the use of blockchain [52], and the fact that not all the patients are interested in managing their health records [9].

Beck and Müller-Bloch [19] suggested that in order to manage a radical innovation with blockchain, 3 competencies are needed to realize its benefits: discovery, incubation, and acceleration. Discovery refers to recognizing and articulating the blockchain opportunities and building research communities. Incubation involves designing blockchain use cases and experimenting with them (ie, proof of concept). Acceleration involves proposing the blockchain implementation and investing in the implementation of a full-functioning blockchain logic and infrastructure. The proof of concept of blockchain technologies strives to replicate real-world conditions in order to evaluate the feasibility of blockchain in health care and address its challenges [8,12]. Even though the required improvements are tested and have provided successful results, they come at the expense of other important aspects in the health information system. A proof of concept for a blockchain-based patient-centric information exchange between patients and providers has provided promising results; however, the real-world implementation is expected to provide different results [53]. Using blockchain to improve health data exchange and patient engagement can come at the expense of performance due to the dynamic regeneration of smart contracts. Additionally, the experimentation files will never be at the same size as the actual patient data [53]. Data size is considered to be one of the important considerations for the feasibility of blockchain solutions for health care [12].

Approaches to implement blockchain in the health care sector can be evolutionary or revolutionary. The evolutionary approach involves integrating blockchain with legacy electronic health records systems, which can compromise the availability of patient information and cause the relaxing of security countermeasures [14]. The revolutionary approach is a bottom-up approach that aims at building the entire health care information system as a blockchain-enabled system and then migrating to it [14]. Both approaches create uncertainties around the cost of implementing or integrating blockchain-based solutions for health care and provide unclear returns on investment. It has been claimed that implementing blockchain-based solutions negatively affects the financial metrics in the short term but pays off in the long term [41]. Reducing operating costs is an important consideration to test the feasibility of blockchain solutions [12].

With blockchain technology, transactions are processed and verified by an automated programmable logic with predefined rules, which reduces transaction costs (ie, effort and time spent on bureaucracy) [17,54]. The consensus mechanism of blockchain ensures the integrity of the data, but calculating the hashes for a single block in the chain is time-consuming and energy-consuming [13,25,32]. Consequently, complex or computation-intense systems are not the best use cases for blockchain [18]. For health care information systems, performance, real-time communication, coordination, data sharing, and medical service availability are critical in life-threatening situations [55].

Some of the major challenges with the current health information systems are interoperability, integration complexities, and the inability of current legacy systems to communicate directly and share health records [40,56,57]. However, the use of blockchain in health care is found to have interoperability challenges, and blockchain, as a radical innovation, brings integration and implementation complexities [9,10,19]. Even though there are proof-of-concept suggestions and experiments to improve the interoperability, the challenges still exist [8]. This implies an unclear difference between no-blockchain and blockchain situations.

### Security Implications of Blockchain in Health Care

It is claimed that private permissioned blockchain deployment brings the most benefits for health care applications [8,9]; however, it brings security risks at the same time [11,41]. Private permissioned blockchains are limited to trusted and predefined participants, and a central authority manages the rights to read and write operations of the blockchain [27]. This feature provides more control by assuring that only authorized participants can perform read or write operations on the patient data [9]. This has positive implications for the confidentiality and integrity of the data. Additionally, the immutability enables tracking of patient-generated data for medical research purposes, transactions on insurance claim processes to detect fraud, and pharmaceutical supply chains for quality assurance [11,41]. Private permissioned blockchain can also enable the availability of audit trails and progress traceability.

In the case of using patient-generated health data for research purposes, smart contracts enable patients to give consent and permission for researchers to access their health data [58]. However, data integrity can be compromised, as the patient data entry point, which is the patient’s device, can be used to impersonate the patient [59]. Sharing patient health data with researchers poses a threat to the privacy of the patient; even if the data are pseudonymized, there is a risk of reidentification [60]. However, the attempts to enhance patient privacy in blockchain environments and design blockchain features for privacy are still in the pilot phase, and there is no guarantee they will preserve privacy [60,61].

Private permissioned blockchains are most prone to a 51% attack [21]. This happens when the central trustworthy node is compromised by the attacker; since the validation of the transactions is centralized, the attacker gains the authority to control the computational power of the network, causing a transaction to happen twice. Hence, the integrity of the transaction data is affected and the resources of the network are exhausted. This has negative implications on the integrity of the data and service availability, which are critical for health care applications [11].

Private permissioned blockchains have limitations in saving patient data with transaction data for the purpose of preventing distributed denial-of-service (DDoS) attacks [12]. This represents an obstacle, as the volume of patient health data is growing over time [12]. Addressing the data size limitation in private blockchain would need to accommodate the increasing volume of patient data, exposing the network to DDoS attacks [12]. Additionally, validating a block of a large data size consumes much power and entails further operational costs [12]. In either case, the service availability, which is critical for health care services, would be compromised.

The security of patient health data with blockchain technology is still in its proof-of-concept phase, and security and privacy are not fully guaranteed so far. The attempts to address security and privacy of blockchain in health care appear to be at the expense of other important features of blockchain technology itself or the needs of the health care sector [62].

## Discussion of the Implications

In this viewpoint paper, we have examined the various innovation and security implications of using blockchain technology in the health care sector. Based on that, we revisit our research question: What implications may blockchain technology bring to the health care sector?

Blockchain technology is not new; however, exploring the feasibility of blockchain applications for the health care sector is in its infancy. The current state of innovating with blockchain in health care is in the proof-of-concept phase [8,58]. Blockchain is a technological innovation that brings benefits and challenges [42]. The health care sector is expected to benefit from blockchain in terms of empowering patients and increasing immutability and traceability [11,41,58]. The needs of the health care sector include sharing vast amounts of patient health data across involved entities (ie, interoperability), regulatory compliance (eg, GDPR), data confidentiality, data integrity, privacy, and data and service availability. The feasibility of using blockchain in health care is dependent on the capability of storing and processing vast amounts of patient health data, ensuring privacy, and reducing operating costs [12,46]. Customizing private permissioned blockchain solutions to fit the needs of the health care sector may result in manipulating the characteristics of blockchain technology [15] or manipulating the needs of the health care sector [12,46]. This is a known trade-off approach happening in the proof-of-concept attempts to apply blockchain solutions in health care [62]. This trade-off involves compromising between two desirable but incompatible features. For instance, complying with GDPR requirements involves manipulating the immutability of blockchain. Additionally, the block size limitation in blockchain is intended to reduce performance overhead and prevent DDoS attacks. However, this compromises the scalability needed to accommodate the vast amount of patient health data [12], and manipulating the size of patient health data is difficult. On the other hand, if the blockchain is designed to process large data, it will cause extra operating costs due to the performance overhead, and it will expose the network to DDoS attacks.

We see the need to distinguish between the benefits and challenges that are unique to blockchain and those that are common across other technological innovations. For example, interoperability is not a challenge specific to blockchain per se; rather, it is a common challenge when adopting any technological innovation.

Based on the topics and views debated in this paper, we summarize the implications of blockchain for health care in terms of both the patients and the health care providers (see Table 1).

**Table 1:** Blockchain implications for the health care sector.

Group	Benefits	Challenges
Patients	Patients are empowered with self-sovereignty through self-managing personal patient-generated health data The identity of the patients is anonymized	Some patients may not be interested in self-managing their health data
Health care providers	Providing a decentralized database with identical copies of the same complete health information, which is made accessible to all parties in the health care chain Facilitating collaboration and data sharing Claimed immutability of a transaction’s history	Interoperability is a challenge, and complex systems are not the best use cases for blockchain DDoS^a^ attacks are likely to happen and affect the availability of the patient health data A 51% attack, specific to blockchain, affects the integrity of transactions’ data and consumes the network resources Compliance issues with GDPR^b^ Blockchain can be resource consuming when all entities in the chain have to approve a large-sized data block

^a^DDoS: distributed denial-of-service.

^b^GDPR: General Data Protection Regulation.

We argue that blockchain technology is surrounded by a controversy between marketing hype and realistic criticism. The marketing hype has manifested in the claim that blockchain is immune to common security attacks that threaten data confidentiality, integrity, and availability. Meanwhile, there are realistic criticisms that show that blockchains are hackable in many ways [21]. A comprehensive list of security threats in blockchain and their causes has already been rendered [29]. This serves as an incentive for research on security improvements for blockchain in general as well as in health care [14,21].

In terms of medical research, there is potential for using blockchain technology. It is possible to store participants’ informed consent to ensure a more transparent, traceable, and tamperproof research method for medical research [63].

This work has implications for further research. Health care researchers can benefit from anonymized health data, which can be shared and aggregated to generate new insights into improving patient health or health care services while maintaining the privacy of patients. Further research is needed to increase awareness about blockchain and to clear the misconceptions and the hype around it. More context-specific use cases need to be designed to avoid generic arguments about blockchain’s applicability across sectors. Future research efforts can aid health care providers in developing the required competencies to innovate with blockchain (ie, discovery, incubation, and acceleration) [19].

## Conclusion

Throughout this paper, we have presented and discussed various views on blockchain technology and the positive and negative issues related to it. Blockchain technology is regarded as a promising technology for securely sharing health data. However, it is not clear if blockchain is really the solution to all the issues regarding highly sensitive data.

Throughout this work, we have highlighted the myths and important challenges concerning blockchain technology. Further, we have questioned the applicability of blockchain technology to the health care sector. Governments may want to examine feasible scenarios in which to use blockchain in the health care sector as well as the challenges associated with the traditionalism of such a sector and the immaturity of blockchain. This requires a careful consideration of the trade-offs that may be made when designing and implementing blockchain solutions for health care.

In this paper, we identified blockchain technology’s positive and negative implications for patients and health care providers, which opens up unlimited opportunities for future research to delve into.

## Abbreviations

DDoS: distributed denial-of-service
GDPR: General Data Protection Regulation
IT: information technology
